# Spinal Concussion

**Published:** 1890-04

**Authors:** 


SPINAL CONCUSSION: *Surgically considered as a Cause of Spinal Injury, and Neurologically restricted to a certain Symptom Group, for which is suggested the designation,* ERICHSEN'S DISEASE, *as one form of the Traumatic Neuroses.* By S. V. Clevenger, M. D., etc. Philadelphia and London: F. A. Davis, 1889.

In view of the fact that the remote effects of spinal concussion may be worse than the immediate, and that much may be at stake in such cases, it behooves every physician to familiarize himself with what is known on this subject, that he may recognize the possible future condition. This book, with its illustrative cases from practice, adds to what has been known of the subject. Erichsen's labors in this field have stimulated the author to the production of this work, which we heartily commend to our readers.

